# Atypical central neurocytoma with leptomeningeal dissemination: a case report

**DOI:** 10.1186/s43046-020-00030-8

**Published:** 2020-05-27

**Authors:** Shikha Goyal, Tejinder Kataria, Deepak Gupta, Aruj Dhyani, Ishani Mohapatra, Karanjit Singh Narang

**Affiliations:** 1 Division of Radiation Oncology, Medanta The Medicity, Gurugram, Haryana 122001 India; 2 Department of Pathology, Medanta The Medicity, Gurugram, Haryana 122001 India; 3 Division of Neurosurgery, Medanta The Medicity, Gurugram, Haryana 122001 India

**Keywords:** Atypical neurocytoma, Craniospinal irradiation, Neuronal tumors, MIB-1 labeling index, Case report

## Abstract

**Background:**

Central neurocytomas represent 0.25–0.5% of all intracranial tumors in adults. Leptomeningeal spread is uncommon, and the exact incidence of meningeal spread is unknown due to sparse literature. We present the clinical course and management outcome of a case of atypical central neurocytoma with leptomeningeal spread.

**Case presentation:**

A young gentleman, who initially presented with memory loss, was found to have a right intra-axial periventricular mass on imaging. He underwent subtotal resection, and operative histopathology suggested a periventricular atypical neurocytoma. In view of subtotal resection, adjuvant focal radiation therapy was recommended, but he developed headache and blurring of vision 10 days postoperatively. Contrast enhanced craniospinal magnetic resonance imaging (MRI) showed residual primary tumor as well as diffuse leptomeningeal spread. Cerebrospinal fluid cytology also showed malignant cells. After tumor board discussion, craniospinal axis irradiation was advised and delivered. He remained disease-free for 10 months after radiation therapy, but then developed local and spinal recurrence, and offered salvage chemotherapy. His general condition deteriorated following chemotherapy with disease progression, and he was subsequently advised best supportive care.

**Conclusion:**

Leptomeningeal dissemination in atypical neurocytomas portends an aggressive course and adverse prognosis; management decisions may need tailoring as per individual presentation.

## Background

Central neurocytomas (CN) represent 0.25–0.5% of all intracranial tumors in adults, mostly affecting patients in third decade and are even more uncommon in children [1]. In their first description of CN in 1982, Hassoun et al. characterized two tumors that were neuronal on electron microscopy but resembled oligodendroglioma rather than medulloblastoma on light microscopy [2]. The authors emphasized on the relatively mature appearing neuronal population of tumor cells and the benign clinical course.

However, subsequent reports of CN described a subset with a more aggressive clinical course. Neurocytoma classification included both typical and atypical types. The most promising marker for predicting tumor aggressiveness is Ki-67/MIB-1 labeling index (LI) and various cutoffs ranging from 2–10% have been proposed [3–5]. The exact incidence of meningeal spread is not known, with only about 20 cases reported in literature, but a recent analysis of atypical neurocytoma with malignant behavior revealed their increased propensity for craniospinal axis dissemination [6]. Juratli et al. have described a case that initially presented with spinal disease at lumbar spine level and misdiagnosed as “atypical” ependymoma. Over a period of 20 years, there were multi-level spinal recurrences managed with surgeries, and only later was a cranial (third ventricular) non-progressive lesion identified [7].

## Case presentation

A 33-year-old gentleman presented to the neurologist with complaints of loss of memory and forgetfulness since 2.5 months, along with recent onset headache and vomiting. His clinical examination showed a Glasgow coma score of 15 with inability to remember recent and some past events and also to perform simple mathematical calculations. There were no appreciable cranial nerve deficits, motor, or sensory loss.

**Fig. 1:**
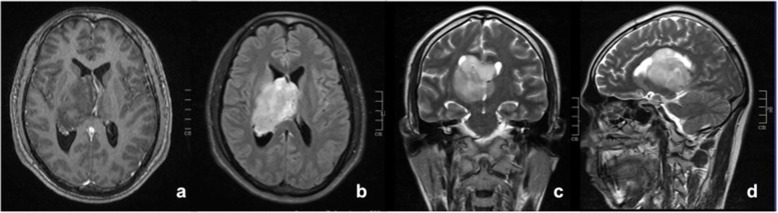
MRI brain (contrast) showed a large, fairly defined lobulated heterogeneously enhancing space occupying lesion with its epicenter in subependymal region of right lateral ventricular body, showing gross extensions into the intraventricular space, thalamus, body of corpus callosum, and leftward displacement of septum pellucidum, with mild ventricular dilatation. There was a small eccentric non-enhancing cystic necrotic focus. A small focus, hypointense on T1 and hyperintense on T2 and FLAIR sequences with restricted diffusion on diffusion-weighted imaging (DWI) and heterogeneous post-contrast enhancement was seen involving left thalamus. No leptomeningeal enhancement was seen. **a** Axial T1 contrast sequence. **b**–**d** Axial, coronal, and sagittal T2 FLAIR sequences of the brain showing the tumor extensions

A contrast enhanced magnetic resonance imaging (MRI) of brain showed a large, fairly defined lobulated heterogeneously enhancing space occupying lesion (SOL) with its epicenter in subependymal region of right lateral ventricular body, showing gross extensions into the intraventricular space, thalamus, body of corpus callosum, and leftward displacement of septum pellucidum. There was mild ventricular dilatation. There was a small eccentric non-enhancing cystic necrotic focus. A small focus, hypointense on T1 and hyperintense on T2 and FLAIR sequences with restricted diffusion on diffusion-weighted imaging (DWI) and heterogeneous post-contrast enhancement was seen involving left thalamus. No leptomeningeal enhancement was seen on the brain imaging, and thus spine was not imaged preoperatively nor was a cerebrospinal fluid (CSF) study performed (Fig. 1). On magnetic resonance spectroscopy (MRS), the lesion showed a high choline to n-acetylaspartate (NAA) ratio (> 2). Findings suggested a mitotic lesion, possibly lymphoma or glioblastoma.

A neurosurgeon was consulted, and only a subtotal tumor excision was possible. Intraoperatively, it was a greyish, soft, moderately vascular, suckable tumor extending inside the frontal horn, atrium, and trigone of right lateral ventricle and to the opposite ventricle.

**Fig. 2:**
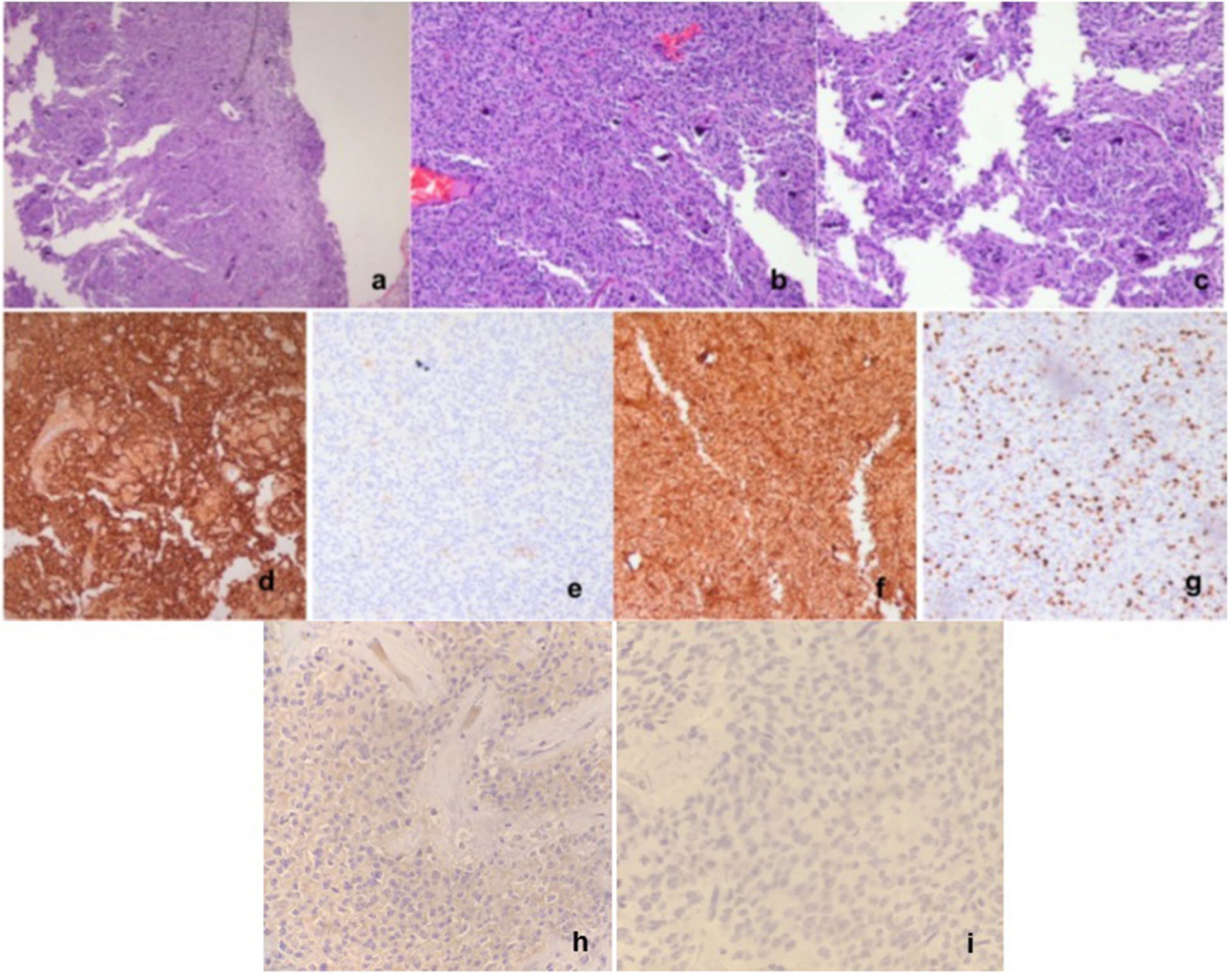
**a** Photomicrographs showing sections of tumor tissue composed of sheets of oligodendrogioma-like cells with round to oval nuclei within a clear cytoplasm in a delicate vascular network, intervening neutrophils, calcification, and Homer-Wright Rosettes (H&E, 4×). **b**, **c** High power views (H&E, 10×) showing necrosis, microvascular proliferation, several mitoses, and areas of hemorrhage. **d** Immunostaining showing diffuse positivity for synaptophysin. **e** Tumor cells are immunonegative for epithelial membrane antigen (EMA). **f** Focal positivity seen for glial fibrillary acid protein (GFAP). **g** Ki-67 labeling index was 8–10%. **h** Isocitrate dehydrogenase 1 (IDH-1) and **i** chromogranin were negative on immunostaining. The morphological and immunohistochemical features are those of an atypical neurocytoma

Histopathological examination showed sections of tumor tissue composed of sheets of oligodendrogioma-like cells in a delicate vascular network, intervening neutrophils, calcification, and Homer-Wright Rosettes. Necrosis and microvascular proliferation were observed. Several mitoses were discernible with areas of hemorrhage (Fig. 2a–c) Tumor cells were negative for isocitrate dehydrogenase 1 (IDH-1) and chromogranin, positive for synaptophysin, focally for glial fibrillary acid protein (GFAP), and negative for epithelial membrane antigen (EMA) and p53. MIB-1 LI (Ki-67) was 8–10% (Fig. 2d–i). A diagnosis of atypical neurocytoma (World Health Organization, WHO grade II), right ventricle was given (Fig. 2).

**Fig. 3:**
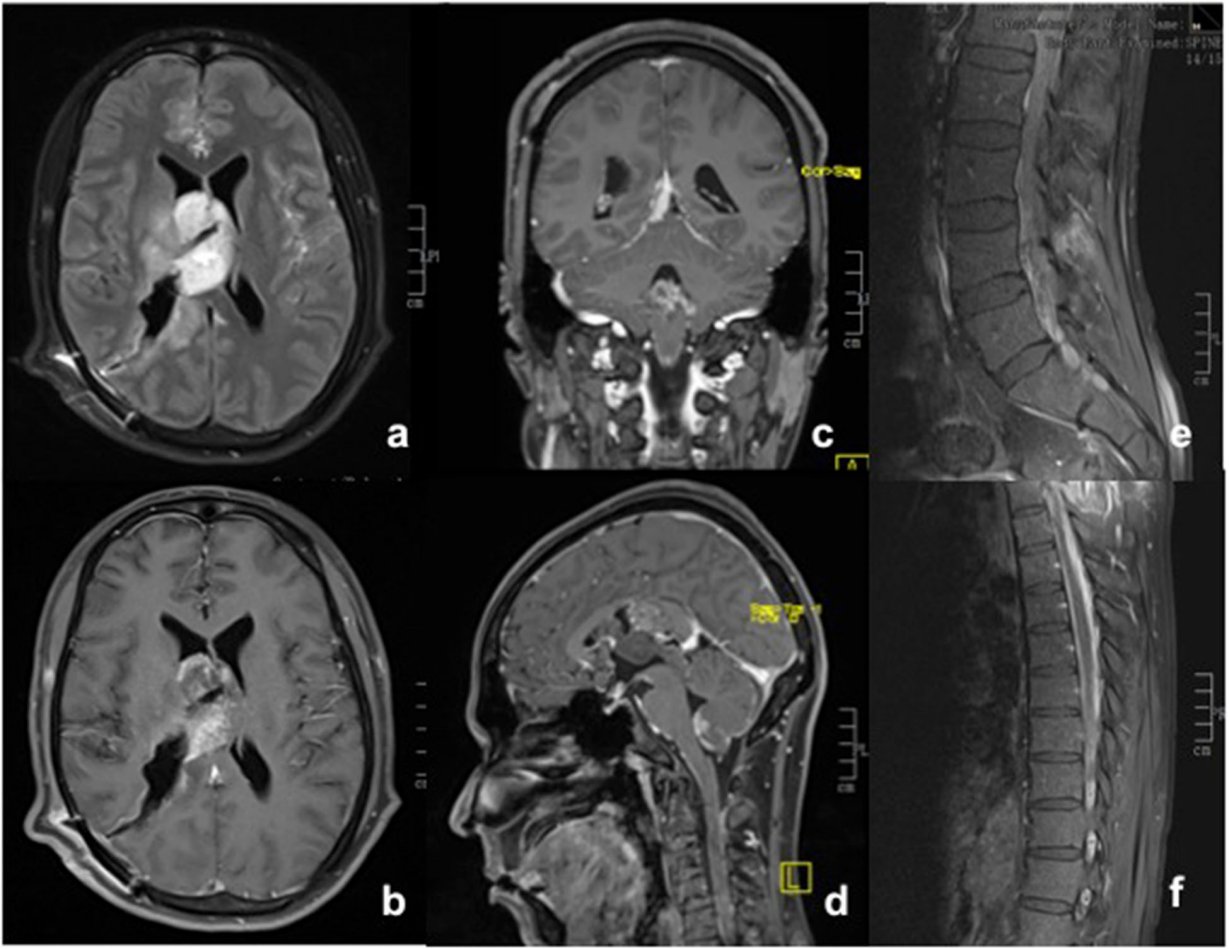
MRI of brain showed the residual right ventricular tumor mass infiltrating into right thalamus, posterior limb of right internal capsule, and reaching across the midline into the medial part of the left thalamus, causing mass effect over the third ventricle and mild upstream dilatation of the lateral ventricles. Abnormal enhancement was seen along the interpenduncular cistern, prepontine, perimesencephalic basal cistern, Sylvian fissures, cortical sulci, floor of fourth ventricle, and tentorium cerebelli, suggesting leptomeningeal spread. **a** T2 FLAIR axial section. **b**–**d** T1 contrast axial, coronal, and sagittal images. **e**, **f** Diffuse leptomeningeal enhancement along pial surface of spinal cord and along the nerve roots of cauda equina without any focal intramedullary lesion

Owing to subtotal excision and high Ki-67, he was advised adjuvant focal radiotherapy (RT) to residual tumor. However, on the tenth postoperative day, he developed sudden onset severe headache, vomiting, and blurring of vision. MRI of brain was repeated along with a screening study of spine with contrast. Residual right ventricular tumor mass was seen infiltrating into right thalamus, posterior limb of right internal capsule, and reaching across the midline into the medial part of the left thalamus, causing mass effect over the third ventricle and mild upstream dilatation of the lateral ventricles. There was abnormal enhancement along the interpenduncular cistern, prepontine, perimesencephalic basal cistern, Sylvian fissures, cortical sulci, floor of fourth ventricle, and tentorium cerebelli, suggesting leptomeningeal spread. Diffuse leptomeningeal enhancement was also seen along pial surface of spinal cord and along the nerve roots of cauda equina. There was no focal intramedullary lesion (Fig. 3). CSF cytology returned positive for malignant cells. A diagnosis of atypical neurocytoma with leptomeningeal spread was made.

**Fig. 4:**
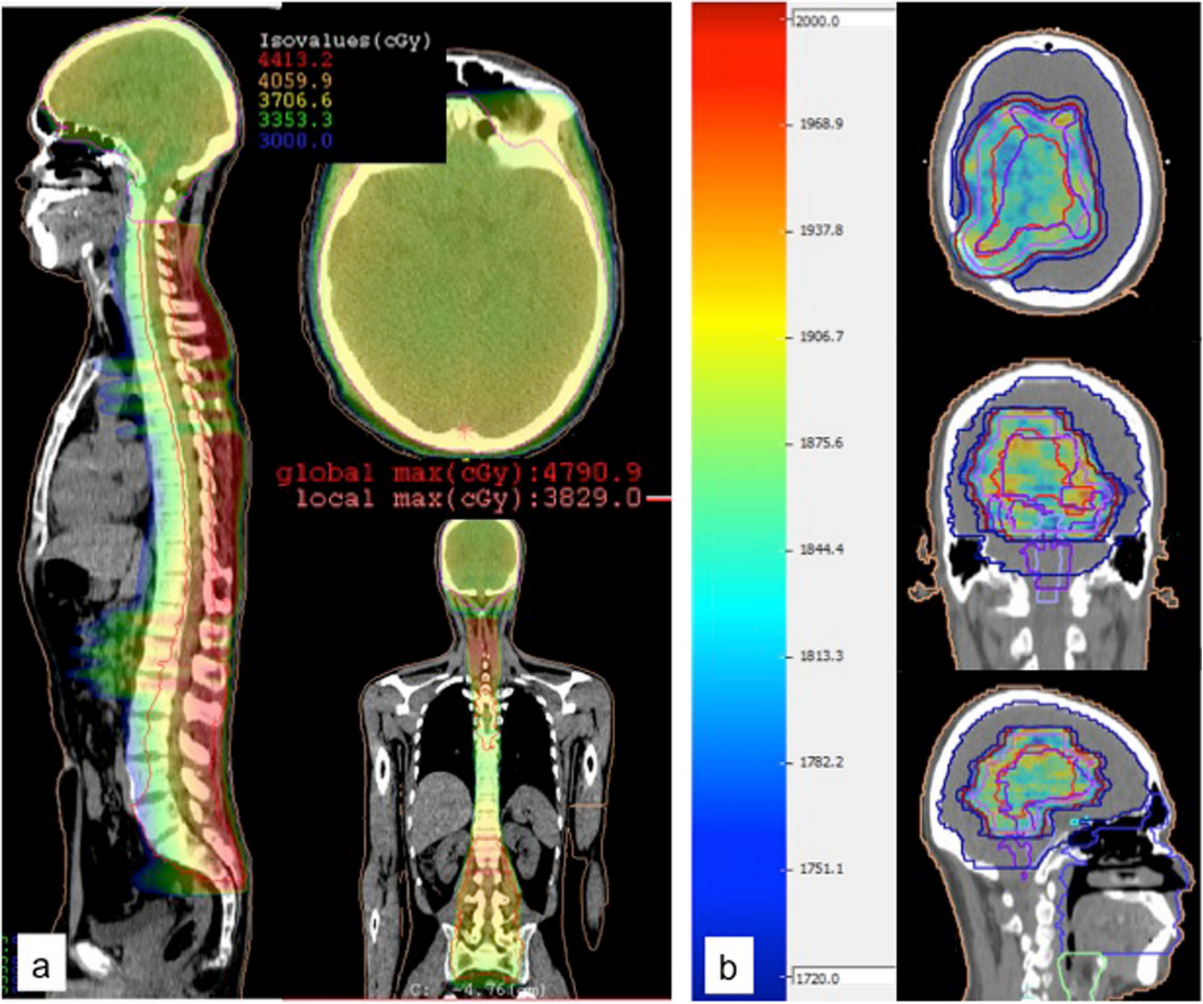
(**a**) Axial, Coronal and Sagittal images of the Craniospinal RT plan showing dose colorwash. A dose of 36 Gy in 20 fractions was planned to the entire craniospinal axis using 3DCRT. (**b**) This was followed by a boost to tumor bed (20 Gy in 10 fractions) using VMAT

The clinical developments were discussed in the multidisciplinary tumor board, and thereafter, the treatment plan was revised. He received craniospinal irradiation (CSI) of 36 gray (Gy) using three-dimentional conformal radiotherapy (3D-CRT) with a tumor bed boost of 20 Gy using volumetric modulated arc therapy (VMAT) (Fig. 4). He completed the planned treatment course with one instance each of grade 2 neutropenia requiring growth factor support, grade 2 mucositis, and grade 1 nausea/vomiting.

**Fig. 5:**
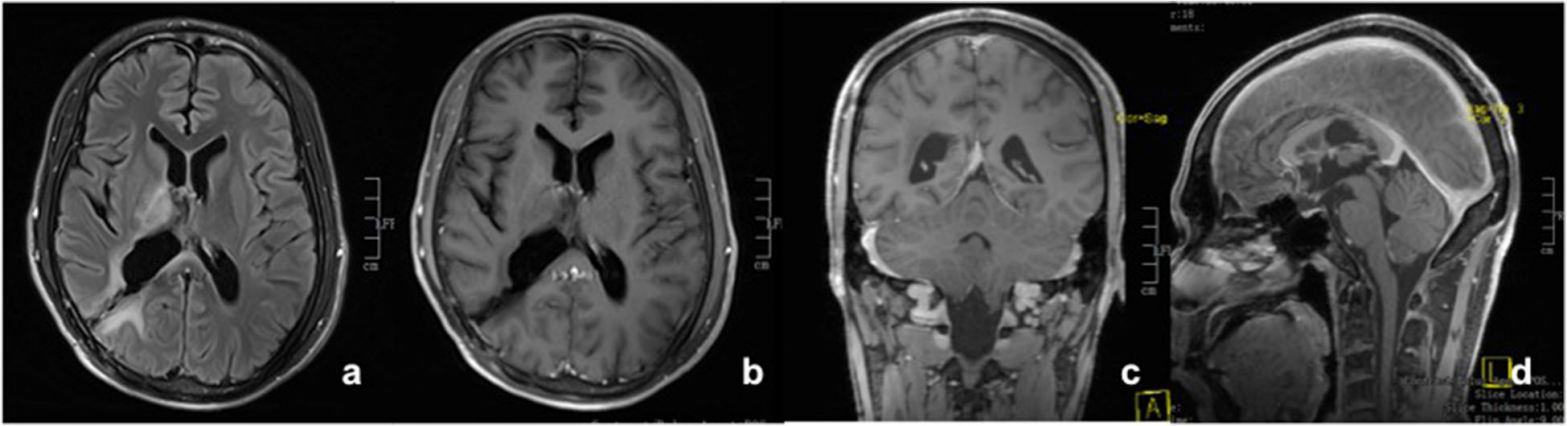
**a** Axial T2 FLAIR and **b**–**d** axial, coronal, and sagittal sections on T1 contrast MRI of brain showing altered signal involving right thalamus, posterior limb of internal capsule (reduced compared to pre-radiotherapy scans) with no abnormal enhancement in this region. Subtle persistent signal alteration in the medial left thalamus. Mild persistent focal leptomeningeal enhancement seen in right side of superior cerebellar and quadrigeminal plate cistern. No residual enhancing mass lesion in the right lateral ventricle or corpus callosum

**Fig. 6:**
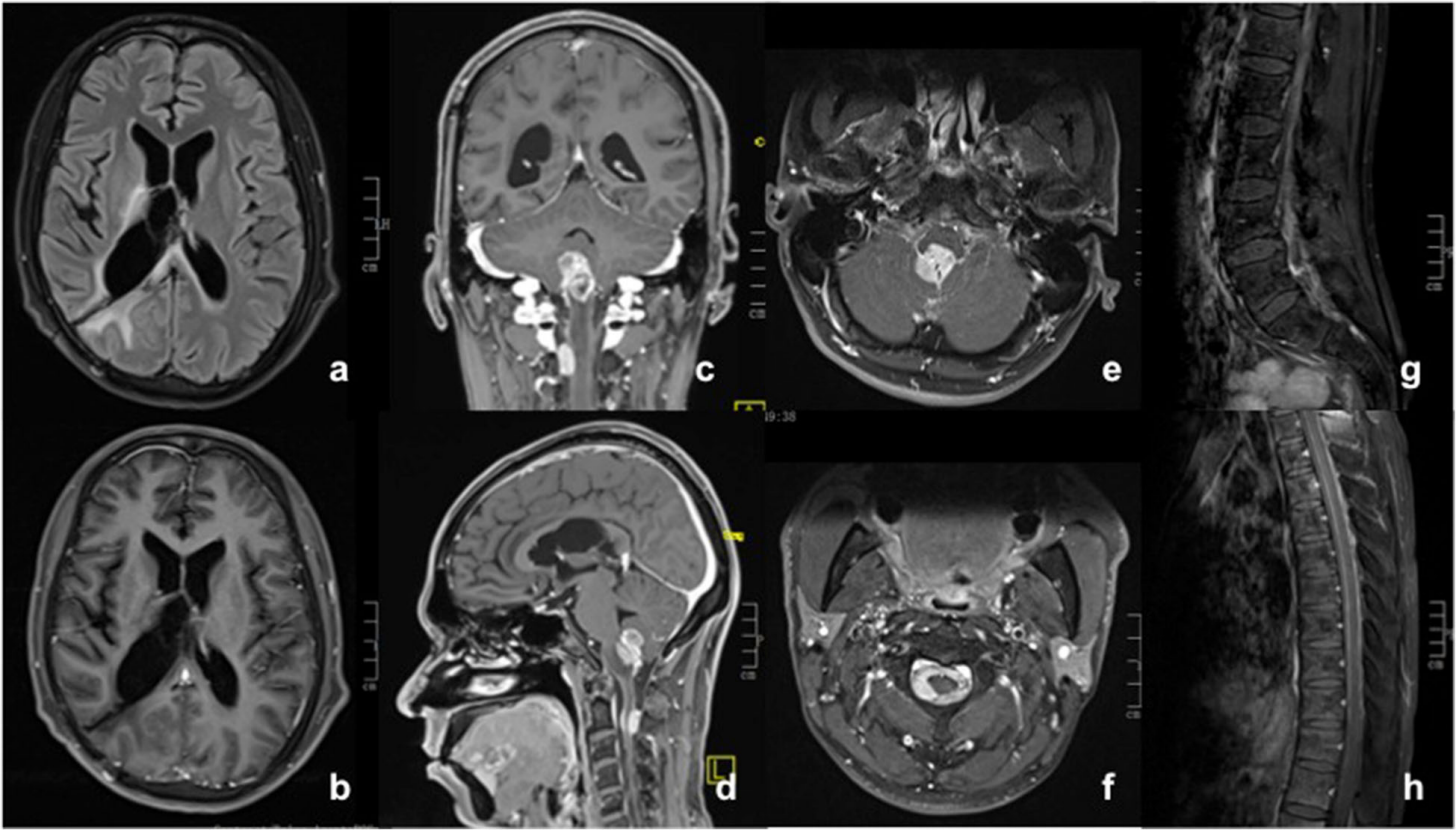
Axial **a** T2 FLAIR and **b** T1 contrast sections of brain showing near complete resolution of the primary lesion. **c**–**f** There is interval development of heterogeneously enhancing mass lesion (2.4 × 2.0 × 1.4 cm) in the inferior part of fourth ventricle causing marked compression on dorsal medulla. Another heterogeneously enhancing nodular lesion (1.1 × 0.6 × 1.4 cm) seen in right posterior subarachnoid space at the level of C2 vertebra causing mild compression of the posterior cervical spinal cord. Abnormal leptomeningeal enhancement is seen along the surface of brainstem and cerebellum. **g**, **h** Abnormal leptomeningeal enhancement is also seen along the surface of entire spinal cord with thickening and clumping of the cauda equina nerve roots

He was on regular follow-up with imaging every 3 months. He was clinically stable but had persistent memory deficits. Partial response in the thalamic lesion and complete resolution of leptomeningeal enhancement were noted on MRI done at 3 and 6 months (Fig. 5). After 10 months of completing CSI, he complained of gait imbalance and tendency to fall on right side along with hand tremors while writing. Contrast MRI of brain and spine showed interval development of nodular mass lesions in fourth ventricular outlet and right-sided posterior subarachnoid space in upper cervical cord at the level of C2 vertebra, along with abnormal leptomeningeal enhancement along ventricles and spinal canal. (Fig. 6). He received 6 cycles of salvage chemotherapy (cisplatin and etoposide every 3 weeks) but his condition deteriorated rapidly thereafter, and after tumor board discussion, he was offered best supportive care.

## Discussion

Most CNs present with features of increased intracranial tension (headache, vomiting), though visual field defects, gait abnormalities, and memory changes may also develop. Clinical suspicion of CN is rare, and the final diagnosis is confirmed only with postoperative histopathology.

Tumors arise from subependymal plate of lateral ventricles. Computed tomography (CT) typically shows a ventricular space occupying lesion, usually involving lateral ventricles; the mass is well circumscribed with attachment to septum pellucidum and enhances with iodinated contrast. Approximately 50% tumors have calcification. MRI shows a tumor that is iso- or hyperintense to cerebral cortex and enhances with contrast. Usually no draining vein is seen, and the attachments and infiltration are better visualized on MRI than on CT. Role of positron emission tomography (PET) or spectroscopy is uncertain [8].

On light microscopy, typical CNs appear as small round cells with round nuclei and scant cytoplasm resembling oligodendroglioma or ependymoma and often stain positive for synaptophysin. Electron microscopy and immunohistochemistry suggest a neuronal origin. Most cases have a favorable prognosis with benign biological behavior. However, atypical aspects have been reported in 20–25% cases [9]. This rare subgroup, termed “atypical central neurocytomas”, constitues tumors with non-central location (extraventricular or spinal), older age at presentation, MIB-1 LI over 2–3%, and/or histologic atypia with infiltrative margins, increased mitoses, necrosis, endothelial or vascular proliferation or cellular pleomorphism, and biological aggression manifesting as early recurrence and progression [3, 5, 9]. A higher cutoff for MIB-1 LI (10%) has been suggested by Qiu-lin et al., with a proposal to label them as “anaplastic neurocytoma, WHO grade III” [4].

Surgical excision is the mainstay of treatment. Gross total resection (GTR) yields a local control (LC) of 57% and 5-year survival (5y-S) of 93% without need for adjuvant treatment. For subtotal resection (STR) alone, LC and 5y-S drop to 7% and 43%, improving to 70% and 78%, respectively, with adjuvant radiation therapy [10].

Rades and Schild, after an extensive review of 438 patients, including 365 adults and 87 atypical CNs, with a minimum follow up of 1 year, gave treatment recommendations for various CN subgroups. No adjuvant therapy is required after GTR. After STR, adjuvant radiation improves survival in atypical lesions and in adults but not in children, while local control improves across all subgroups. Adults need doses exceeding 54 Gy while doses above or below 50 Gy have comparable outcomes in children. Authors recommended adjuvant RT doses of 50 Gy in children, 50–54 Gy in typical CN, and 56–60 Gy in atypical CN [11]. Stereotactic radiosurgery with peripheral doses of 9–25 Gy (mean 14.9 Gy) yields comparable control rates but slightly lower incidence of complications and higher risk of distant failures, though the difference is not significant [12].

A literature review of 19 atypical cases with malignant behavior showed MIB-1 LI above 2% in 12 of 14 cases with available data and above 10% in those progressing within a year [6]. Seventeen patients had craniospinal dissemination though CSI was given to 3 patients only after documented craniospinal dissemination. The documented disease-free follow-up information for these 3 cases was available at 3 months, 3 years, and 5.5 years, respectively [13–15]. In their own case, the Mozes et al. considered CSI upfront but owing to lack of robust supporting literature, delivered it only after the patient developed spinal dissemination at 3 years. They recommended that CN cases with high malignant potential should be considered for maximal tumor resection followed by adjuvant CSI, instead of only adjuvant local RT [6]. Leenstra et al. reported a 35-year experience of 45 cases, wherein 3 patients were given prophylactic CSI (2 after resection and 1 after biopsy) with CSI doses of 30–36 Gy and tumor bed boost of 20 Gy, describing long survival exceeding 7 years in one of their cases [16].

Role of chemotherapy is uncertain and has been explored in inoperable or disseminated cases, with reports of stable disease and partial response with regimens including carboplatin, vincristine, topotecan, cyclophosphamide, and temozolomide [6, 13, 17, 18]. Stem cell transplantation following high dose chemotherapy has also been tried [19].

In the present case, there was no clinical evidence of CSF dissemination at diagnosis but appeared soon after surgery, suggesting an aggressive behavior. Although CSI was given in adjuvant setting, he remained relapse-free for only 10 months, indicating that perhaps adjuvant chemotherapy should also have been considered. Additionally, we recommend spinal screening for all cases with a MIB-1 LI of > 4% or several features of atypia such as necrosis, mitoses, or vascular proliferation on microscopy.

### Patient’s perspective

The patient belonged to the lower socioeconomic group. The diagnosis of malignancy at a young age was disheartening; but despite seeking medical attention in time and timely surgery, the course of disease was unpredictable and hard to manage due to mounting side effects of treatment as well as financial challenge for cost of interventions, lodging of family members away from home and loss of work as well as bleak future prospects.

## Conclusion

Atypical central neurocytoma may occasionally exhibit aggressive behavior. Treatment may need to be tailored to specific presentation, imaging, and pathologic findings.

## Data Availability

All data generated or analyzed during this study are included in this submitted article.

## Abbreviations

CN: Central neurocytoma
MIB-1 LI: MIB-1 labeling index
MRI: Magnetic resonance imaging
SOL: Space occupying lesion
DWI: Diffusion-weighted imaging
CSF: Cerebrospinal fluid
MRS: Magnetic resonance spectroscopy
NAA: n-acetylaspartate
GFAP: Glial fibrillary acid protein
EMA: Epithelial membrane antigen
WHO: World Health Organization
RT: Radiotherapy
CSI: Craniospinal irradiation
Gy: Gray
3D-CRT: Three-dimentional conformal radiotherapy
VMAT: Volumetric modulated arc therapy
CT: Computed tomography
PET: Positron emission tomography
GTR: Gross total resection
LC: Local control
IDH-1: Isocitrate dehydrogenase 1
